# The Functional Networks of Prepulse Inhibition: Neuronal Connectivity Analysis Based on FDG-PET in Awake and Unrestrained Rats

**DOI:** 10.3389/fnbeh.2016.00148

**Published:** 2016-07-21

**Authors:** Cathrin Rohleder, Dirk Wiedermann, Bernd Neumaier, Alexander Drzezga, Lars Timmermann, Rudolf Graf, F. Markus Leweke, Heike Endepols

**Affiliations:** ^1^Department of Psychiatry and Psychotherapy, Central Institute of Mental Health, Medical Faculty Mannheim, Heidelberg UniversityMannheim, Germany; ^2^Institute of Radiochemistry and Experimental Molecular Imaging, University Hospital of CologneCologne, Germany; ^3^In-Vivo NMR Laboratory, Max-Planck-Institute for Metabolism ResearchCologne, Germany; ^4^Forschungszentrum Jülich GmbH, Institute of Neurosciences and Medicine, INM-5: Nuclear ChemistryJülich, Germany; ^5^Department of Nuclear Medicine, University Hospital of CologneCologne, Germany; ^6^Department of Neurology, University Hospital of CologneCologne, Germany; ^7^Multimodal Imaging of Brain Metabolism, Max-Planck-Institute for Metabolism ResearchCologne, Germany

**Keywords:** behavioral PET, metabolic connectivity, PPI network, PPI modulation, cerebral glucose consumption

## Abstract

Prepulse inhibition (PPI) is a neuropsychological process during which a weak sensory stimulus (“prepulse”) attenuates the motor response (“startle reaction”) to a subsequent strong startling stimulus. It is measured as a surrogate marker of sensorimotor gating in patients suffering from neuropsychological diseases such as schizophrenia, as well as in corresponding animal models. A variety of studies has shown that PPI of the acoustical startle reaction comprises three brain circuitries for: (i) startle mediation, (ii) PPI mediation, and (iii) modulation of PPI mediation. While anatomical connections and information flow in the startle and PPI mediation pathways are well known, spatial and temporal interactions of the numerous regions involved in PPI modulation are incompletely understood. We therefore combined [^18^F]fluoro-2-deoxyglucose positron-emission-tomography (FDG-PET) with PPI and resting state control paradigms in awake rats. A battery of subtractive, correlative as well as seed-based functional connectivity analyses revealed a default mode-like network (DMN) active during resting state only. Furthermore, two functional networks were observed during PPI: Metabolic activity in the lateral circuitry was positively correlated with PPI effectiveness and involved the auditory system and emotional regions. The medial network was negatively correlated with PPI effectiveness, i.e., associated with startle, and recruited a spatial/cognitive network. Our study provides evidence for two distinct neuronal networks, whose continuous interplay determines PPI effectiveness in rats, probably by either protecting the prepulse or facilitating startle processing. Discovering similar networks affected in neuropsychological disorders may help to better understand mechanisms of sensorimotor gating deficits and provide new perspectives for therapeutic strategies.

## Introduction

Prepulse inhibition (PPI) of the acoustic startle response is a measure of sensorimotor gating, and implies a reduction of the startle response, when a non-startling stimulus (prepulse) is presented within a certain time frame before the startling stimulus (Hoffman and Searle, 1967; Hoffman and Ison, 1980). Since PPI can be studied across species (Swerdlow et al., 1999) and impairments are observed in several neuropsychological diseases (for review see Braff et al., 2001; Kohl et al., 2013), deficient PPI is deemed as an endophenotype for such disorders. Brain areas mediating startle and PPI are located within the brain stem and include cochlear nuclei, caudal pontine reticular nucleus, inferior and superior colliculus, pedunculopontine and laterodorsal tegmental nuclei as well as substantia nigra (for review see Fendt et al., 2001; Swerdlow et al., 2001). In addition, several cortico-limbic areas modulate PPI-mediation, like nucleus accumbens, ventral pallidum, basolateral amygdala, septohippocampal system, mediodorsal thalamus, and medial prefrontal cortex (for review see Swerdlow et al., 2001; Koch and Fendt, 2003). Although deficient PPI modulation is associated with symptom severity in schizophrenia (Hazlett et al., 2007), little is known about the exact function of the modulation network. We hypothesized that it continuously adjusts sensorimotor gating, since it is active even if no selective attention to prepulse or startle stimuli is required (Rohleder et al., 2014). In order to examine its functional importance and specifically the interplay between PPI-modulating brain areas, we performed functional connectivity analysis with the PET images described in Rohleder et al. (2014), as this methodological approach allows delineating distinct networks associated with different aspects of the behavioral paradigm.

In animal research, functional neural network analysis is so far focused on networks activated under resting state conditions, and is based on analysis of temporal correlations of low-frequency (<0.1 Hz) blood-oxygenated level dependent (BOLD) signal fluctuations (Biswal et al., 1995; Schwarz et al., 2013a), measured by functional magnetic resonance imaging (fMRI). If an individual is awake and alert but not engaged in an attention-demanding situation, particular brain areas are tonically active and form a functional network that is termed default mode network (DMN; Gusnard and Raichle, 2001; Raichle et al., 2001). Several studies have analyzed this network in anesthetized, sedated or awake but fixated animals (Liang et al., 2011; Upadhyay et al., 2011; Lu et al., 2012; Wehrl et al., 2013, 2014). It is very likely, that anesthesia, sedation or restraint stress influence the results and limit the analyzable behavioral spectrum.

An approach to overcome this problem is positron emission tomography (PET) using the tracer [^18^F]fluoro-2-deoxyglucose (FDG), which provides information on cerebral glucose consumption (Dienel and Cruz, 2008) related to neuronal activity (Kornblum et al., 2000). Behavioral FDG-PET (bFDG-PET) allows for temporal separation of radioligand accumulation during a behavioral task (e.g., PPI) and the subsequent scan that requires anesthesia (Rohleder et al., 2014). Advantages are that firstly neither FDG accumulation nor the actual PET measurement induces restraint stress. Secondly, functional network analysis is not only restricted to DMN but can be expanded to functional networks relevant for specific behavioral tasks. As proof of principle we visualized the DMN, and then focused on the analysis of functional PPI networks. From previous correlation patterns between FDG uptake and PPI effectiveness (Rohleder et al., 2014), we hypothesized that PPI modulation may be implemented by two distinct networks, as substantiated in the present study.

## Materials and methods

### Subjects

Nineteen healthy, untreated male adult (postnatal day >106, 388 ± 48 g) rats were used. Fourteen of them were Black hooded rats (Janvier, France), five were Lister hooded (Charles River, Germany). Both originate from the same rat strain of the Lister Institute, but Lister hooded rats derived from outbred, Black hooded from inbred breeding. Lister hooded rats possess higher hearing thresholds, resulting in less PPI events, since PPI depends on salience against background (for details see Rohleder et al., 2014). Lister hooded rats are less sensitive not only to the startle stimulus but also to the prepulses resulting in a different signal-to-noise ratio compared to Black hooded rats. Therefore, including both rat strains into the analysis allows obtaining a wider range of behavioral data. Animals were housed together in pairs in type 4 cages enriched with a horizontal tube for climbing and a nest box under controlled ambient conditions (22 ± 1°C and 55 ± 5% rh) on an inversed 12-h light/dark schedule (lights on 8:30 p.m.–8:30 a.m.). Rats had free access to water, but diet was restricted. All experiments took place during the dark, i.e., active phase of the rats' day-night cycle.

Experiments were carried out according to the German law on animal protection and approved by the local animal care committee (State Office for Nature, Environment and Consumer Protection of North-Rhine Westphalia, Dept. Animal Welfare).

### Experimental design

In order to screen for gross structural anomalies and to facilitate coregistration of PET images, MRI was accomplished. Subsequently, two different bFDG-PET experiments were conducted on separate days. In a counterbalanced order either a background control (resting state) or a PPI paradigm was combined with PET. Intra-individual difference images of both behavioral paradigms were used for further seed-based network analysis.

MRI, PET, and PPI paradigm have been previously described in detail (Rohleder et al., 2014) and are only briefly summarized in the following.

### Magnetic resonance imaging (MRI)

T2-weighted, rapid acquisition with relaxation enhancement (RARE) images were recorded with an 11.7-T BioSpec animal scanner (Bruker BioSpin® MRI, Ettlingen, Germany) by using a quadrature receive-only rat brain surface coil (Bruker BioSpin®) in combination with an actively decoupled, transmit-only quadrature resonator (Bruker BioSpin®), fitting into the BFG-150/90-S14 combined gradient and shim set (Resonance Research Inc., MRI, Ettlingen, Germany). During the procedure inhalation anesthesia was used (initial dosage: 5% isoflurane in O_2_/N_2_O (3:7), reduced to 1.5–2.5% isoflurane for maintenance).

### Behavioral pet imaging (bFDG-PET)

Three minutes subsequent to an i.p. injection of 500–700 μl [^18^F]fluoro-2-deoxyglucose solution (FDG, ~2 mCi), rats underwent a behavioral paradigm (details see below) for 45 min. After each session the enclosure was cleaned with diluted acetic acid. During the behavioral paradigm, FDG accumulated in energy-consuming brain cells. Afterwards, rats were anesthetized, placed, and fixed on an animal holder (medres®, Cologne, Germany) with a respiratory mask, whereby inhalation anesthesia procedures were similar to those used during MRI scans. Static PET scans were performed using a Focus 220 micro PET scanner (CTI-Siemens®). The 30 min data acquisition period started exactly 1 h after FDG-injection. Following Fourier rebinning, data were reconstructed using the iterative OSEM3D/MAP procedure (Qi et al., 1998) resulting in voxel sizes of 0.38 × 0.38 × 0.82 mm.

#### Prepulse inhibition (PPI) paradigm

The PPI paradigm took place in the SR-Lab (San Diego Instruments®, San Diego, USA). All presented acoustic stimuli (white noise: 2.2–16.7 kHz) had a duration of 25 ms. The 45 min experimental session was composed of a habituation period [1 min background noise followed by 25 startle stimuli with a randomized interstimulus interval of 3–13 s (average interval: 7.6 s)] and the actual PPI paradigm. The latter comprised 300 trials (30 trials per stimulus type) presented in a pseudorandomized order with a randomized interstimulus interval of 3–13 s (average: 7.6 s). The trials included the following stimulus types: (a) control trials (background noise: 65 dB SPL LIN), (b) startle-alone trials (110 dB SPL LIN), (c) prepulse-alone trials (68, 72, 78, or 84 dB SPL LIN), and (d) PPI trials where startle pulses were preceded by 68, 72, 78, or 84 dB SPL LIN prepulses. The interval between prepulse and startle was 100 ms (onset to onset).

Startle amplitude was measured by a piezoelectric accelerometer below the tubular enclosure and calculated as integrated response of the whole body startle reaction over 100 ms, starting 5 ms after startle stimulus onset (recording range: 5–105 ms). All values were baseline corrected.

PPI was calculated for each prepulse intensity as percent reduction of the average startle amplitude (A):

$$\begin{array}{l} PPI\;\left[\%\right]=\left(A_{\text{startle alone}}-A_{\text{prepulse}+\text{startle}}\right)\;\times \;100∕A_{\text{startle alone}} \end{array}$$

Startle events were defined as A_startle alone_ > 30 mV or A_prepulse + startle_ with PPI < 15%. PPI events were defined as A_prepulse + startle_ with PPI > 15% (Geyer and Swerdlow, 1998). In order to obtain a standardized measure of PPI effectiveness, the number of startle and PPI events was determined for each animal, and the relative difference was calculated:

$$\begin{array}{l} \text{PPI effectiveness }\left[\%\right]=\left({\text{n}}_{\text{PPI}}-{\text{n}}_{\text{startle}}\right)\times 100∕\left({\text{n}}_{\text{PPI}}+{\text{n}}_{\text{startle}}\right) \end{array}$$

Negative values indicate more startle, whereas positive values signify more PPI incidents.

The use of four different prepulse intensities results in a functional continuum that ensures maximal flexibility of behavior. This allows analyzing the underlying brain mechanisms of physiological instead of unnatural extreme behaviors.

#### Background noise control (resting state)

For background noise control, i.e., resting state measurements, rats were placed for 45 min into the tubular enclosure of the SR-Lab, but were exposed solely to continuous background noise (65 dB SPL LIN).

### Data analysis

Imaging data were analyzed using the imaging software tool VINCI 4.35 (Vollmar et al., 2007). MR images that were matched to a standardized rat brain atlas (Swanson, 1998) served as anatomical templates, and facilitated coregistration of PET images. Assignment and designation of brain areas were based on the brain atlas of Paxinos and Watson (2005). Subsequently, PET image intensities were normalized by the use of the ratio normalization technique (Arndt et al., 1996). For that purpose the olfactory bulb was chosen as reference area (Rohleder et al., 2014) since its activity was supposed to be similar in both conditions (rats were both times exposed to the smell of diluted acetic acid).

**Figure 1:**
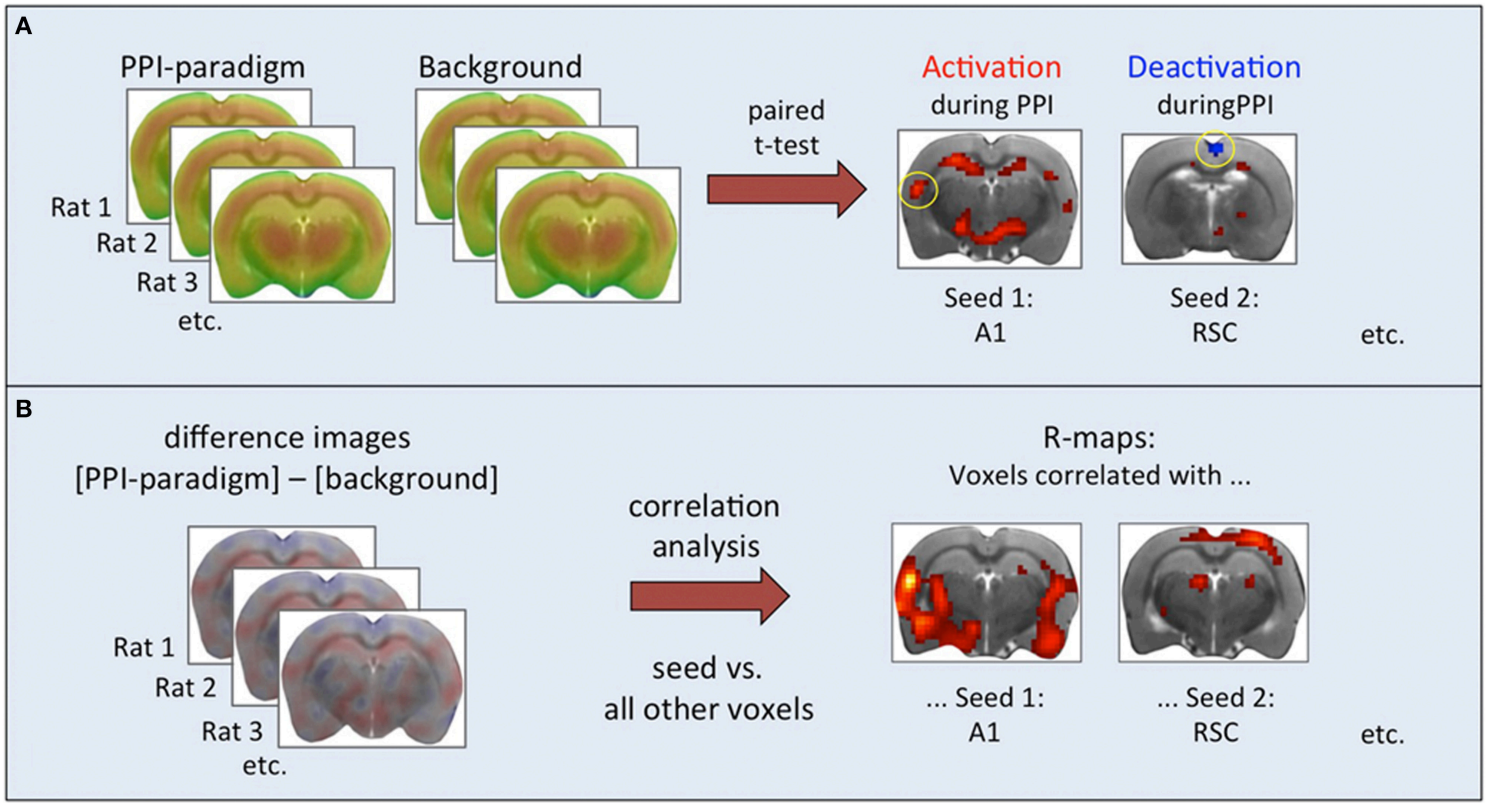
**Steps of functional connectivity analysis. (A)** Paired *t*-test based on individual pairs of cumulative FDG images. Results were used to select seed regions. **(B)** Correlation analysis based on intra-individual difference images. Associations between seed regions and all other brain voxels were calculated and displayed as correlation maps thresholded at *p* < 0.01 (TFCE-corrected).

For each of the 19 animals, two FDG-PET images were available: one taken during the PPI session, and one during the background noise session. A paired *t*-test was performed to visualize regions of activation and deactivation during the PPI session compared to the background noise session (Figure 1A). The Pearson product-moment correlation test was used to assess the relationship between intra-individual difference images (PPI paradigm minus background) and PPI effectiveness. As seeds we chose two areas which were activated during PPI (right CuN/PPTg and left A1), one area deactivated during PPI (RSC), one region positively correlated to PPI effectiveness (right PrL), and a negatively correlated one (VTA; see Table 1).

**Table 1:** **Seed overview**.

**Seed region**	**Activity during PPI paradigm vs. resting state**	**Correlation with PPI effectiveness**
RSC	Deactivated	No significant correlation
CuN/PPTg (right)	Activated	No significant correlation
VTA	Activated	Negative
A1 (left)	Activated	No significant correlation
PrL (right)	No significant change	Positive

*A1, primary auditory cortex; CuN, cuneiform nucleus; PPTg, pedunculopontine tegmental nucleus; PrL, prelimbic cortex; RSC, retrosplenial dysgranular cortex; VTA, ventral tegmental area*.

Pearson correlation analyses were performed with the Gauss-filtered (1.5 mm FWHM) intra-individual difference images (PPI paradigm minus background) across all animals, comparing the seed regions with all other brain voxels (Figure 1B). Significant positive correlations indicated that the respective voxels took up FDG analog to the seed region, i.e., in animals where the seed region had a high FDG uptake, positively correlated voxels had a high FDG uptake as well, and vice versa. Significant negative correlations were not found.

To correct for multiple testing with 18,450 brain voxels, t-maps underwent threshold-free cluster enhancement (TFCE, Smith and Nichols, 2009) and subsequent permutation testing. The TFCE procedure takes the height of the individual voxel *t*-score as well as the cluster size into account and is based on the following equation:

$$\begin{array}{l} TFCE\left(\upsilon \right)=\int_{0}^{t\left(\upsilon \right)}h^{H}{e\left(h\right)}^{E}dh\approx \underset{0,\delta ,2\delta ,\ldots ,t\left(\upsilon \right)}{\sum }h^{H}{e\left(h\right)}^{E}\delta \end{array}$$

with *t(*ν*)*: *t*-value of the voxel ν; *h*: height, incrementally raised from zero up to *t(*ν*)* in steps of δ = 0.1; e: cluster extent (number of voxels). Exponents *H* = 2 and *E* = 0.5 were empirically determined by Smith and Nichols (2009).

TFCE-values were corrected at the level of *p* < 0.01 via permutation testing: Values of the seed area and the voxel with the maximum TFCE score were extracted from the input data (i.e., the intra-individual difference images; PPI paradigm minus background). One thousand permutations were used to generate the null distribution of the t statistic for the correlation coefficient. The TFCE image was thresholded at the 99th percentile of the null distribution.

## Results

### Comparison between PPI paradigm and background

**Figure 2:**
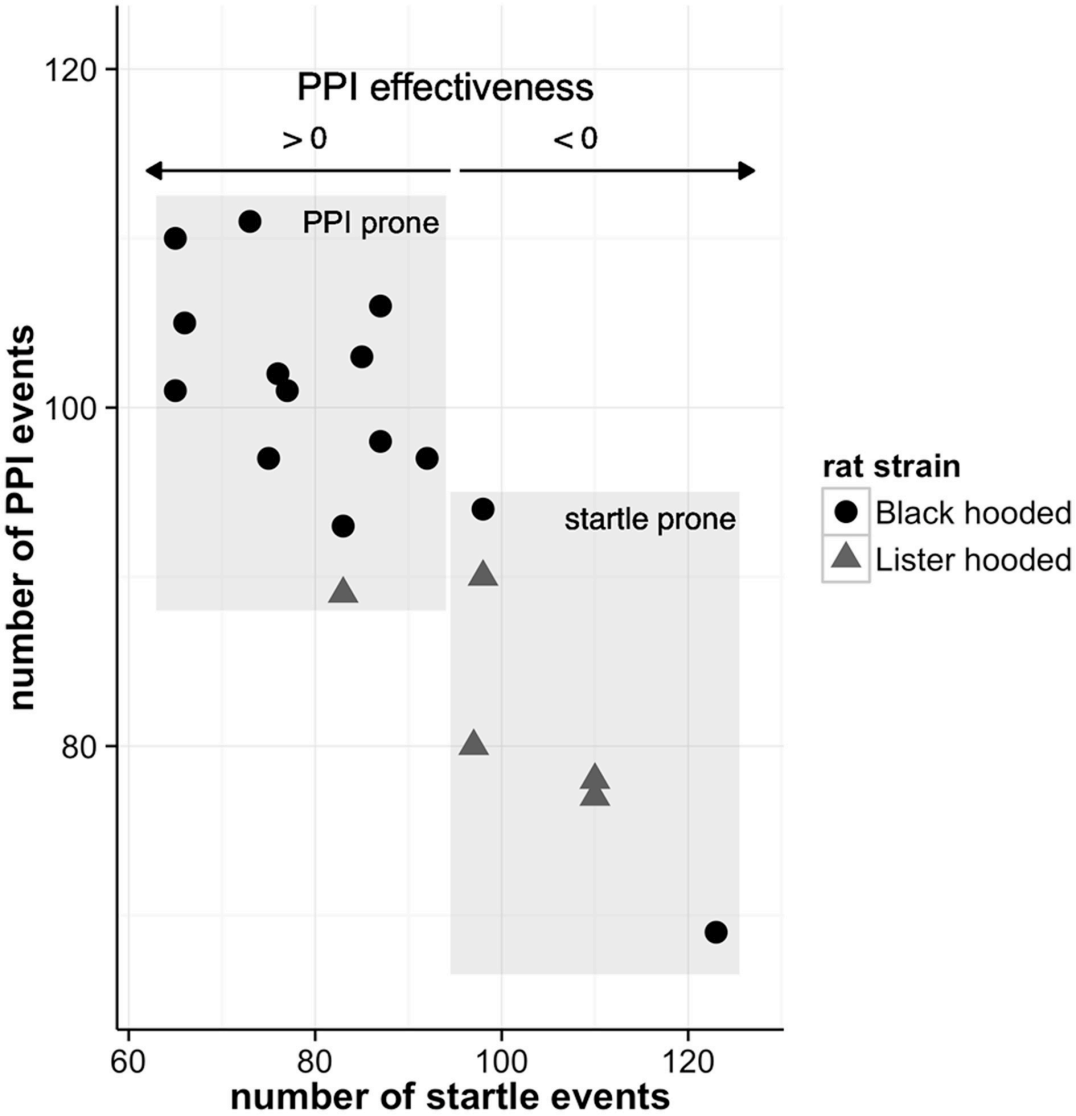
**Individual PPI effectiveness**. The PPI effectiveness resembles the relative difference of the number of PPI and startle events. If animals experience more PPI than startle events, PPI effectiveness is greater than zero whereas negative values signify more startle events. While most of the well-hearing Black hooded rats are more prone to PPI, the Lister hooded rats exhibit higher hearing thresholds and are prone to startle (for hearing threshold details see Rohleder et al., 2014).

As aforementioned, the neural substrates of the PPI can be divided into three brain circuitries: (i) a primary startle pathway mediating the startle reaction (ii) a PPI mediation network, which is responsible for the startle amplitude reduction, when a startle stimulus is preceded by a non-startling stimulus, and (iii) a PPI modulation network, regulating PPI by influencing the PPI mediation circuitry. During the PPI paradigm, the animals were exposed to altogether 345 stimuli: 75 startle-alone, 120 startle stimuli paired with prepulses, and 150 prepulse-alone or control trials. The animals effectively startled 87 ± 16 times (range: 65–123) per session, while the number of PPI events (i.e., reduction of startle amplitude of more than 15%) was 95 ± 12 (range: 69–111). We can therefore assume that both, startle- and PPI-related networks, were active in each animal. The individual PPI effectiveness of the rats is depicted in Figure 2.

**Figure 3:**
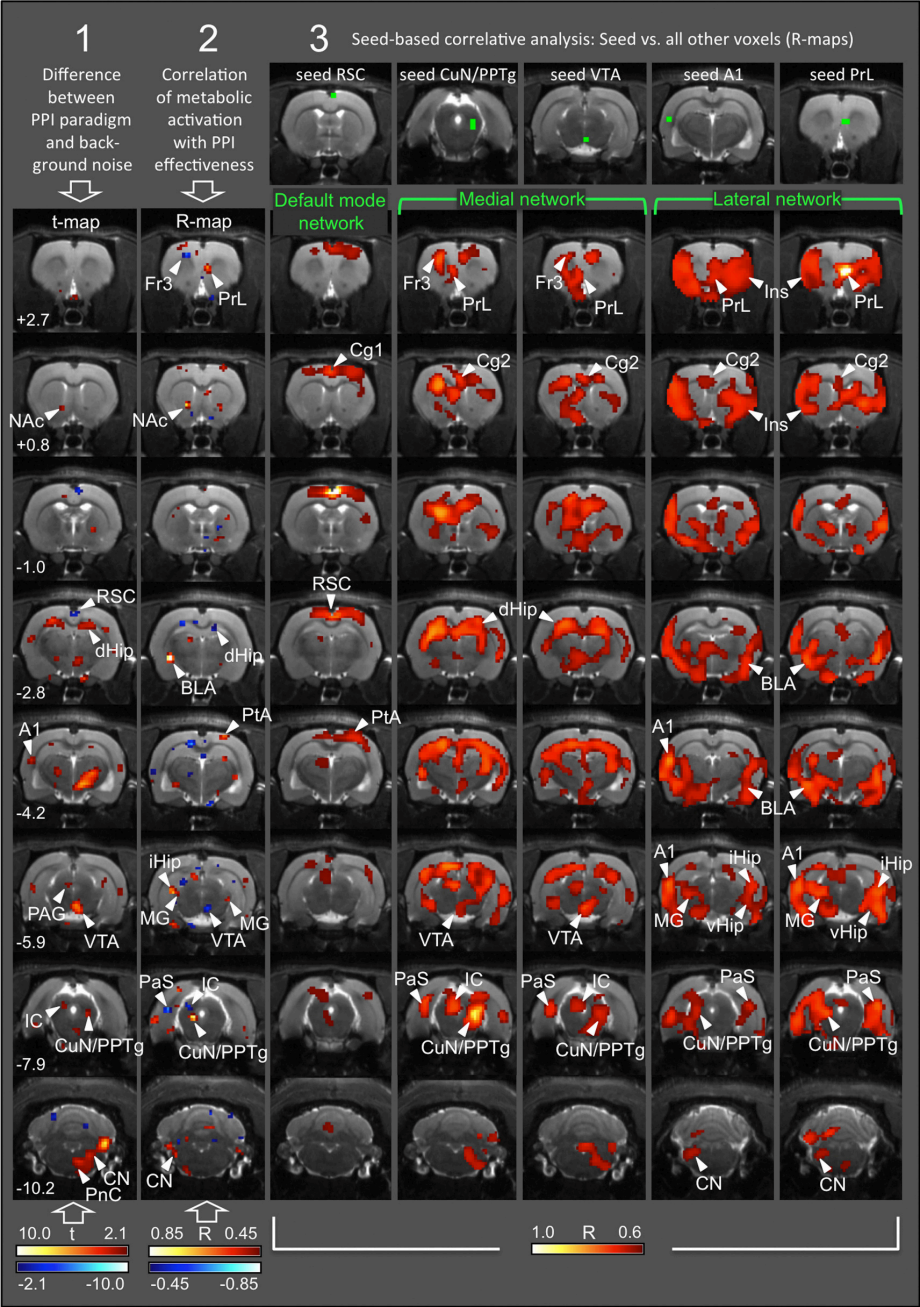
**Functional connectivity networks arising during PPI**. Each column represents a single analysis, with statistical maps projected onto transverse MR images from one of the rats used in this study. Numbers (rows) correspond to rostrocaudal coordinates in mm from Bregma. Column 1: t-map from comparison of FDG uptake between PPI and background sessions, based on *n* = 19 pairs of individual cumulative images. Red, FDG uptake higher during PPI; blue, FDG uptake higher during background control. Only significant differences (*p* < 0.05, uncorrected) are shown. Column 2: R-map from correlation analysis between intra-individual difference images (background minus PPI; *n* = 19) and PPI effectiveness. Red, the more PPI events, the higher the metabolic activity. Blue, the more startle events, the higher the metabolic activity (−0.45 < R >0.45; *p* < 0.05, uncorrected). Columns 3–7: R-maps from correlation analyses between seed area (green voxels; top row) and all other brain voxels, based on *n* = 19 intra-individual difference images. There were significant positive correlations only (*R* > 0.60; *p* < 0.01, TFCE-corrected). A1, primary auditory cortex; BLA, basolateral amygdala; Cg1/2, anterior cingulate nucleus 1/2; CN, cochlear nucleus; CuN, cuneiform nucleus; dHip, dorsal hippocampus; Fr3, frontal cortical area 3; IC, inferior colliculus; iHip, intermediate hippocampus; Ins, insular cortex; MG, medial geniculate nucleus; NAc, nucleus accumbens; PAG, periaqueductal gray; PaS, parasubiculum; PnC, caudal pontine reticular nucleus; PPTg, pedunculopontine tegmental nucleus; PrL, prelimbic cortex; PtA, parietal association cortex; RSC, retrosplenial dysgranular cortex; RtTg, reticulotegmental nucleus of the pons; SC, superior colliculus; SON, superior olivary nucleus; TeA, temporal association cortex; vHip, ventral hippocampus; VP, ventral pallidum; VTA, ventral tegmental area.

Statistical comparison of FDG uptake during the PPI paradigm vs. background noise revealed metabolic activation of the following areas (Figure 3, first column): **Startle pathway**: cochlear nucleus (CN), caudal pontine reticular nucleus (PnC), ventrolateral tegmental nucleus (VLTg; not shown). **PPI mediation network**: ventral inferior colliculus (IC), cuneiform nucleus (CuN), pedunculopontine tegmental nucleus (PPTg), dorsolateral periaqueductal gray (PAG). **PPI modulation network**: ventral tegmental area (VTA), nucleus accumbens (NAc) core, dorsal hippocampus (dHip). In addition, we found increased FDG uptake during the PPI paradigm in the auditory cortex (A1), dorsal hypothalamus, and a large cluster in the thalamus, comprising ventral thalamus and zona incerta. Reduced FDG uptake during the PPI paradigm was found in the anterior part of the retrosplenial dysgranular cortex (RSC).

According to these results, four seed areas were chosen: left A1, right CuN/PPTg, and VTA (significantly higher FDG uptake during the PPI session), as well as right RSC (significantly higher FDG uptake during the background noise session).

### Correlational analysis

#### Metabolic activation and startle behavior

In order to assess which brain areas were associated with the characteristics of startle behavior, we correlated the intra-individual difference images [PPI minus background] with PPI effectiveness (Figure 3, second column see also Rohleder et al., 2014). The correlation clearly illustrates the association between behavioral data and functional connectivity networks as described below. While the prelimbic cortex (PrL), NAc core, basolateral amygdala (BLA), parietal association cortex (PtA), and CuN/PPTg were positively correlated with PPI effectiveness and therefore associated with PPI (red voxels), voxels in the frontal cortical area 3 (Fr3), dHip, parasubiculum (PaS), inferior colliculus (IC), and VTA were negatively correlated with PPI effectiveness, indicating association with startle (blue voxels). From these results we chose a fifth seed area in the right PrL. For seed overview see Table 1.

#### Functional connectivity

Correlative analysis using the five seed regions mentioned above revealed three different networks on the voxel level, defined by significant positive correlations. Significant negative correlations were not found.

- Voxels correlated to the RSC seed comprised the **default mode network** (Figure 3, third column) with the entire frontoparietal cortex, including the anterior cingulate cortex 1 (Cg1), primary motor and sensory areas (M1 and S1), as well as parietal association cortex (PtA). As was to be expected, the default mode network was active during the background noise session, indicated by the higher FDG uptake during background control (blue voxels in Figure 3, first column).
- Voxels correlated to the right CuN/PPTg and VTA seeds, respectively, showed an almost identical pattern corresponding to a **medial network** (Figure 3, columns four and five). This included left Fr3, left PrL, anterior cingulate cortex 2 (Cg2), IC, dHip, and PaS. Interestingly, the left CuN/PPTg was not part of the medial network.
- A **lateral network** emerged with both left A1 and right PrL seeds (Figure 3, column six and seven). It included Cg2, insular cortex (Ins), BLA, ventral hippocampus (vHip), medial geniculate (MG), cochlear nucleus (CN), and left CuN/PPTg.

Notably, the medial network comprised brain areas, which were associated with startle (blue voxels in Figure 3, second column), such as Fr3, dHip, left PaS, IC, and VTA. On the other hand, the lateral network included brain areas associated with PPI (red voxels in Figure 3, second column), such as right PrL, BLA, and left CuN/PPTg. This indicates that the medial network was more active in animals prone to startle, while the lateral network predominated in animals with strong PPI (for an overview of individual PPI effectiveness see Figure 2).

## Discussion

### Functional connectivity analysis with FDG-PET

Glucose metabolism as measured by FDG-PET directly refers to excitatory or inhibitory metabolic brain activity. Functional connectivity assessment with FDG-PET is based on one cumulative metabolic value measured per voxel across a number of animals, rather than on the time course of a physiological signal (e.g., BOLD fluctuations) per voxel in one animal. A similar approach has been followed using [^14^C]iodoantipyrine cerebral blood flow (CBF) autoradiography (Wang et al., 2015). In contrast to this study, which relied on absolute CBF values obtained from one behavioral condition—due to the terminal nature of autoradiography—we have measured the animals in two different settings (PPI session and background noise control). We were therefore able to use intra-individual difference images for correlation analysis, reflecting the metabolic change obtained in the PPI session compared to background noise control. This procedure eliminates variations of regional baseline metabolic activity across animals, which may otherwise obscure metabolic associations between brain areas. Furthermore, reasonable seed areas can be chosen from t-maps displaying significant differences between conditions, which allows analyzing selected networks known to be activated or inhibited during the task of interest.

### DMN-like network

One of the networks we identified in this study was correlated to the seed in retrosplenial cortex, and was suppressed during the PPI session compared to background noise control. It covered the dorsal (parietal) and medial part of the cortex from rostral to caudal pole, but had also subcortical clusters in the thalamus, midbrain, and cerebellum. This network closely resembles the DMN-like connections described in anesthetized (Lu et al., 2012; Schwarz et al., 2013a) and awake rats (Upadhyay et al., 2011). Extracting a DMN-like network from our data provides the proof of principle that seed-based correlative analysis of FDG-PET images allows to identify meaningful functional networks in rats.

### Medial and lateral networks related to PPI and startle processing

**Figure 4:**
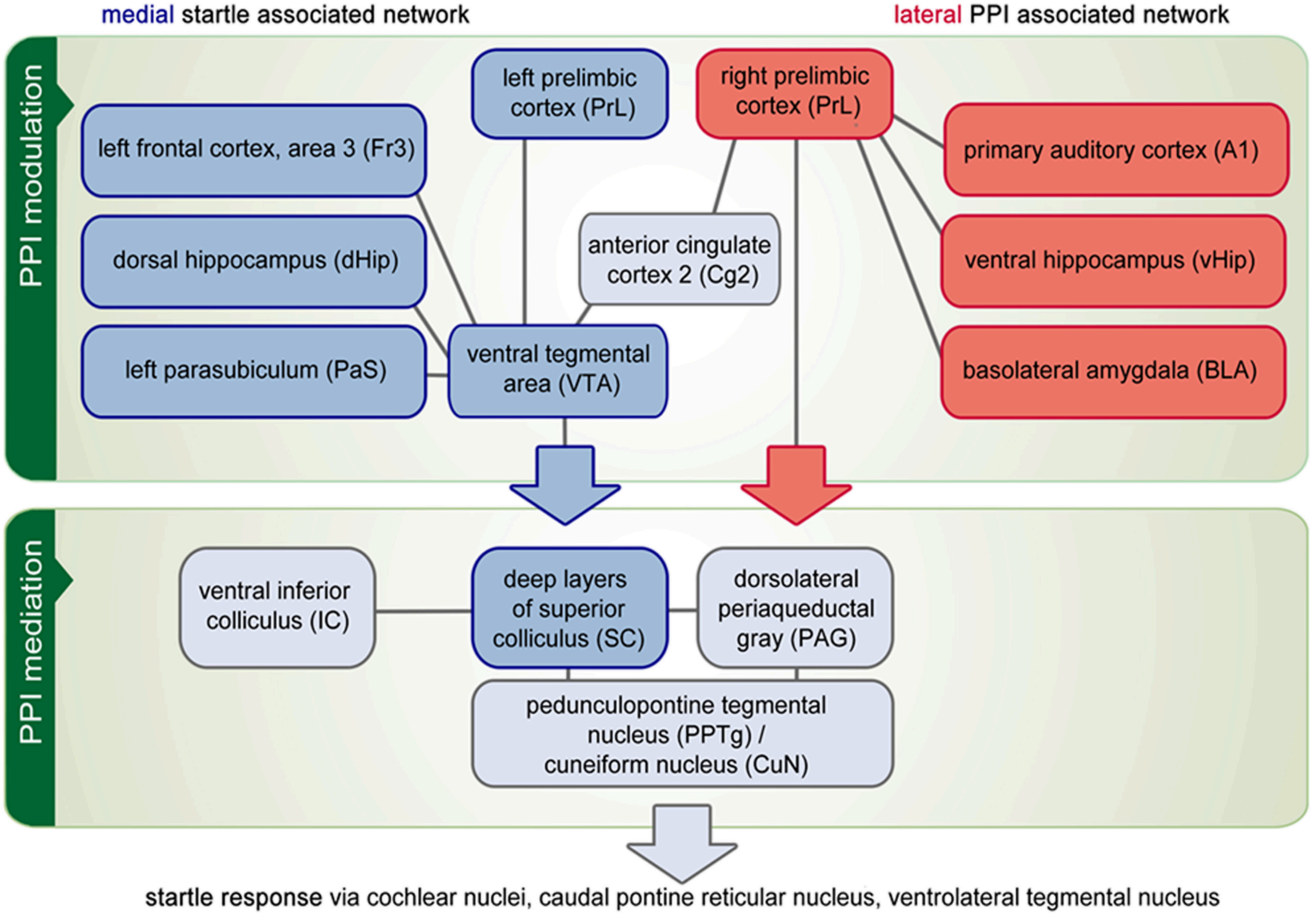
**Functional network analysis**. The PPI of the acoustical startle reaction comprises three brain circuitries: a startle mediation, a PPI mediation and a PPI modulation network. Correlational analysis revealed that above all, the PPI modulation network is composed of a medial part (blue boxes), which correlates to the number of startle events and a lateral network (red boxes) that is activated in correlation to the number of PPI events. On the other hand, some brain areas are part of both the medial and the lateral network (gray boxes). This applies in particular for those areas that constitute the PPI mediation network. It is hypothesized that the PPI modulation network influences the PPI mediation continuously either by protecting prepulse processing (depicted by the red arrow) or by facilitating startle and disrupting prepulse processing (blue arrow). Finally, the activity of the PPI mediation network controls the startle response (gray arrow).

As depicted in Figure 4, two other networks emerged in conjunction with the PPI session: Seeds in the VTA or right CuN/PPTg result in a medial network. In addition to the seed areas themselves, it contained the left PrL, left Fr3, dHip, PaS, and left IC, which were all activated in correlation to the number of startle events (i.e., they were anti-correlated to PPI effectiveness). This medial network comprised most components of the “hippocampal-prefrontal system” described by Schwarz et al. (2013b) and Liang et al. (2011). It is generally thought that the medial network is involved in cognitive and spatial processing (Wang and Cai, 2006).

In contrast, both left A1 and right PrL seeds yielded a lateral network including BLA, vHip, left CuN/PPTg, MG, and CN, which were activated in correlation to PPI effectiveness. The lateral network also covered almost the entire lateral cortex including S1 (jaw region), S2 as well as Ins, which were not directly linked to startle or PPI processing. The lateral network found in this study resembles the “lateral cortical network” reported by Schwarz et al. (2013a). It contains mostly limbic structures (Drevets et al., 2008), conveying emotional information.

During the PPI paradigm, where no problem-solving, decision-making, or other cognitive/executive actions are needed, those networks may be recruited for PPI *modulation*. Their participation in PPI *mediation* is unlikely, because the radius of the PPI mediation circuit is localized within the pons, quite close to the startle circuit. The short startle/PPI latency (around 8 ms in rats) allows maximally five neuronal connections between hair cells and the point at which the returning PPI mediation signal intersects the primary startle pathway (Swerdlow et al., 2001).

It is therefore thought that the PPI modulation network controls sensorimotor gating by influencing the activity of the PPI mediation network continuously (Rohleder et al., 2014), related to attentional and emotional states (Li et al., 2009). Our data suggest that the PPI modulation network consists of two opposing parts, one of which protects processing of the prepulse (“protection hypothesis”; Blumenthal et al., 2015). The other part facilitates startle and interrupts processing of the prepulse (“interruption hypothesis”). Disentangling the involvement of these two distinct networks in neuropsychiatric disorders may promote the development of new therapeutic strategies restoring specific network disturbances.

### The medial network and startle facilitation

It is not surprising that startle facilitation recruits the medial spatial/cognitive network, because in natural threatening situations it is vitally important for the animal not only to prioritize the startling stimulus, but also to memorize its origin and own current location. The dHip contributes to the medial cognitive/spatial circuitry via its connections to midline cortical areas involved in spatial processing (Strange et al., 2014), as well as to the PaS (van Groen and Wyss, 1990), which is responsible to define the animal's own position in space (Taube, 1995; Boccara et al., 2010). The VTA sends dopaminergic projections to all areas of the hippocampal-prefrontal network, which may serve to tonically adjust the startle priority level. This is in line with the wealth of data demonstrating that dopamine agonists disrupt PPI, i.e., facilitate processing of the startle stimulus (Geyer et al., 2001). Medial modulation network and startle pathway seem to interact mostly on the motor side, on the level of the caudal pontine nuclei, as proposed in earlier studies (summarized in Schmajuk and Larrauri, 2005).

### The lateral network and prepulse protection

Our data indicate that the mechanism protecting prepulse processing and preventing interruption by the startle stimulus is based on the lateral emotional network. This is in line with earlier findings (Du et al., 2011) identifying two regions from the lateral network, A1 and the lateral amygdala, as crucial for PPI enhancement by fear conditioning of the prepulse. Our results suggest that those regions are not only active during fear conditioning, but also during classical PPI sessions. At first glance, the involvement of the BLA is contradictory, as a body of evidence point to a crucial modulating role of BLA in fear-conditioning and fear-potentiated startle. Thus it might be assumed that BLA also facilitates startle processing rather than protecting the prepulse. On the other hand it might be suggested that the role of the BLA depends on the threatening level of a situation. For instance it has been hypothesized that in humans mild, distant threat cues may recruit the BLA and the orbitofrontal cortex to enhance fear evaluation and processing and to exert inhibitory regulation as long as fear execution behavior is not yet necessary (Koen et al., 2016). This differential role might be based on the recruitment of different neuron populations. While it has been shown that decreased serotonergic and increased glutamatergic signaling in the BLA is involved in facilitation of fear-potentiated startle (Tran et al., 2013), PPI is modulated by BLA dopamine (Stevenson and Gratton, 2004).

The emotional network may provide its own tonic signal, which continuously adjusts the level of prepulse protection. The participation of higher auditory pathway areas (A1 and MG) and CN in the lateral network indicates that prepulse protection may be accomplished on the sensory side of the startle pathway, by a descending inhibitory circuit (Carlson and Willott, 1998) localized within the auditory system. Corticofugal projections from A1 to the dorsal cochlear nucleus (DCN) have been described in the rat (Weedman and Ryugo, 1996), which may be involved in sound amplitude selectivity. Auditory neurons responding preferentially to low stimuli such as the prepulses, are best described in the DCN (Zhou et al., 2012), but are also present in the cochlear root nucleus (Sinex et al., 2001), the main sensory subregion of startle mediation. Those neurons may trigger PnC inhibition after a prepulse, most likely via the PPI mediation structures IC, SC, and CuN/PPTg (Fendt et al., 2001).

### Connection of medial and lateral network

The medial and lateral networks are coupled to each other within several areas. The Cg2 region from rostral to caudal pole seems to serve as a hub directly connecting the systems. Other regions lateralize with respect to network participation: The medial network recruits the left PrL, left septum, and right CuN/PPTg, while the lateral network contains the right PrL, right septum, and left CuN/PPTg. Although the degree of cerebral lateralization may be variable between individuals (Wang and Liu, 2014), our results nevertheless demonstrate that two counterbalancing networks regulating one behavioral function may use in parallel left- and right-hemispheric parts of one brain area. Another well-described example is the frontoparietal network in humans, which is coupled to the default mode network in the left hemisphere and to the attention networks in the right hemisphere (Wang et al., 2014).

### Conclusion

Our study provides evidence that PPI mediation is modulated by the interplay of two distinct functional networks. The lateral network involves the auditory system as well as the emotional network and seems to protect prepulse processing and hence increases PPI. The medial network recruits the spatial/cognitive network and appears to facilitate startle processing by focusing on the motor side of the startle pathway. Based on the fact that the startle system responds to multimodal stimuli and serves to protect the head from blows (closing the eyes, overall muscle contraction; Yeomans et al., 2002) and that PPI enables perceptual and motor orienting responses by reducing the sensitivity of the startle system for a few hundred milliseconds (Fendt et al., 2001), the two counterbalancing modulation networks may reflect the weighting of protective and orienting behaviors in order to react most appropriately to potentially harmful stimuli.

On the basis of our results, the next step should be the analysis of functional PPI networks in healthy volunteers and patients. Clinical use may benefit from our findings inasmuch as knowing the affected modulation network in a neuropsychological disorder with disturbed PPI might help to further understand underlying pathomechanisms. For instance functional imaging studies on schizophrenic patients indicate impairment of the lateral network (Kumari et al., 2007). Those deficits are situated in the auditory system (Hirano et al., 2010) as well as in the emotional regions (Kumari et al., 2007). Strengthening of the lateral network, e.g., by more specific pharmacological targeting or even deep brain stimulation, may be a future therapeutic approach to alleviate symptoms related to sensory overload.

## Author contributions

CR, HE, and FML conceived the study with input from AD; CR and HE developed and organized the study with input from FML and RG; BN and DW contributed reagents/materials/analysis tools; CR and HE performed experiments; HE and CR analyzed data and drafted the manuscript with input from LT. All authors contributed to final manuscript preparation, discussed the results and their implications, and have read and approved the final manuscript.

## Funding

Financial support was provided by the German Research Foundation (DFG), clinical research group 219, EN 439/4-1.

### Conflict of interest statement

The authors declare that the research was conducted in the absence of any commercial or financial relationships that could be construed as a potential conflict of interest.
